# Progress in Medicine

**Published:** 1898-10-01

**Authors:** 


### Progress in Medicine.

### DISEASES OF THE LUNGS.

(*Continued from page* 410, *Vol. XXIV.*)

**Prognosis in Phthisis.**—Loomis[8] contributes a very practical paper on the prognosis of phthisis. He says that each case is after all unique, and must be considered in all its personal equations. Prognosis must be reached in every case from consideration both of the constitutional condition of the patient as well as of the lung condition. The most important point in prognosis is the condition of the patient's stomach. If assimilation of food is good, the prognosis is always good, often even with desperate lung conditions; on the other hand it is very bad when, in spite of careful attention to diet and method of feeding, it is impossible to keep the patient's digestive organs in good condition. The second condition which renders the prognosis bad is a rapid heart action, noted at the beginning of a tuberculosis, when there is only slight involvement of the lung. The rapid heart action in these cases is not dependent on fever, for at a time of day when the temperature is normal the pulse is still rapid. Age is not an important element in the prognosis as far as the curability of the disease is concerned. The importance of the fibroid diathesis is often overlooked in prognosis, especially when the disease develops in middle life; in people with this inherited condition, irritations have always a tendency to set up fibroid changes. If this diathesis is present, it is an element of favourable prognosis. Hæmoptysis early in the disease does not affect the prognosis one way or the other, especially if it is not followed by fever, lasting a number of days. Hæmorrhages occurring later in the development of the disease are of much more serious import. Tuberculous heredity has very little to do with the question of a person's recovery, all other things being equal. Alcoholic subjects do badly when they contract phthisis, the resisting power of their tisses seems to be at a minimum. Their course downwards is generally rapid. Loss of flesh is an important element when the patient is placed under suitable climatic conditions. Unless a person is gaining weight he is not improving, no matter which way other symptoms point. It is one of the best tests we have for improvement in phthisis; for it to occur many things must happen of a beneficial character—the fever must diminish or disappear, sleep must be fair, and the sweats must stop. The "personality" of the patient is an important point; the person of strong determined character and happy disposition, who has made up his mind to get well, does so though his disease be advanced. Another patient, of a nervous temperament, easily influenced by surroundings, and depressed by any slight change in his symptoms, hardly ever does well. The pulmonary element, more than any other, which makes prognosis favourable, and arrest and final cure of phthisis possible, is the localisation of the pulmonary lesion. The prognosis is favourable, even though there be a cavity at one apex, when the rest of the lungs is normal; whilst it is unfavourable though the tuberculous change is of the slightest imaginable, when it is scattered here and there. In that large class of patients in whom tuberculosis develops secondarily to a subacute pleurisy, or is co-existent with marked changes in the pleura, favourable climatic conditions do much towards cure, and recovery is the rule rather than the exception.

**Diagnosis of Phthisis.**—The first physical evidence of phthisis is heard at the back, not the front, of the chest. The patient's arm is brought forward and the hand placed on the opposite shoulder, and the stethoscope placed on that part of the lung now uncovered, just above and external to where the bronchial tubes are given off. If phthisis is present there will be heard prolonged tubular breathing and fine rales on coughing will be heard. By this means a diagnosis may be made weeks before the physical signs can be appreciated in front, where they are ordinarily looked for. The physical signs generally antedate any expectoration which can be satisfactorily obtained.

The turberculin test is one of very great accuracy, and yet little used for diagnosis in man, though extensively employed in animals. The method of procedure is as follows: Having taken the patient's temperature for a few days to make sure that he has no diurnal elevation above what is normal, half a milligram of tuberculin is injected. At the end of two days if there has been no rise in temperature, or one of less than a degree, a second injection of two milligrams should follow, and if still no reaction after two days more, one of five milligrams. If there is still no reaction, there is no active tuberculosis present, and the patient has either not got the disease, or if he has had it recovery may be considered to be complete, and he may be allowed to return to work. These injections are absolutely safe when given in the doses indicated. The Röntgen rays have of late been extensively used in the diagnosis of phthisis; they mostly serve as a corroborative agent. They enable us to map out fully and accurately the limits of consolidated areas, and to determine the presence of excavations, conditions which at times it is almost impossible to decide by physical examination. The method, however, necessitates considerable practice.

[8] Loomis, New York Med. Rec., May 21, 1898.

### DISEASES OF CHILDREN.

**Sterno-mastoid Tumours.** — On this subject Mr. Jordan Lloyd[1] writes that two fallacies are still prevalent—firstly, that these tumours are gummata, and secondly, that the children will "grow out of" the wry neck which follows the injury to the sterno-mastoid muscle. The so-called sterno-mastoid tumour is a swelling in the muscle produced by rupture of its fibres during the birth of the child, more especially in cases of breech presentation. The treatment ought to be commenced as soon as the lesion is observed. It is but too often overlooked by nurses and doctors and consists of fixation of the head with the face towards the affected side, so that the muscle does not heal in the shortened position. If untreated, then wry neck, with its sequelæ of symptoms, scoliosis, facial hemiatrophy, &c., is apt to follow. This condition can be relieved only by operation, namely, division of the sterno-mastoid muscle. Full details of the operation are given, Mr. Lloyd preferring the "open method" to subcutaneous division.

**Venesection in Children's Diseases** has been found of value by Professor Baginsky.[2] It proved a life-saving measure in three patients suffering from respectively cardiac disease, cirrhosis of the lung and bronchiectasis, and broncho-pneumonia; but in other cases it has not been so beneficial, possibly from the presence of more serious conditions. He thinks that in eclampsia, whether simple or uræmic, the treatment is specially valuable. In one case of severe uræmic eclampsia, where the patient was cyanosed and pulseless with Cheyne-Stokes respiration, and where douches, chloral hydrate, chloroform, &c., had been tried without benefit, the application of six leeches was followed by improvement, and ultimately by slow recovery.

**A Diagnostic Sign of Measles.**—Dr. Koplik,[3] of New York, has again directed attention to an important sign in the early diagnosis of measles, before the appearance of the typical rash. The author's description of it is as follows: "If we look into the mouth at this period (the stage of invasion) we see in a strong light the usual redness of the fauces; perhaps, not in all cases, a few spots on the soft palate. On the mucous membrane lining the cheeks and lips (buccal mucous membrane) we see a distinct and pathognomonic eruption. This consists of small irregular spots of a bright red colour; in the centre of each spot is the interesting sign to which I wish to call attention. In strong daylight we see a most minute bluish-white speck. Those minute bluish-white specks in the centre of a reddish spot are absolutely pathognomonic of beginning measles." He dwells on the value of this sign in preventing epidemics of the disease in hospitals and institutions where large numbers of children are housed, as by means of it measles can be diagnosed at an earlier stage than has hitherto been possible. Confirmatory evidence as to the diagnostic importance of this buccal eruption is given by Dr. E. Libman[4] from an experience of 50 cases.

**Thymus Hypertrophy and Sudden Death.**—The thymus gland is liable to a condition of simple hypertrophy, which occurs in infants who are otherwise healthy, is frequently unaccompanied by any symptoms, and often terminates in sudden death. The clinical picture as given by Dr. Berthold[5] is a sufficiently striking one. An apparently healthy infant is being fondled by its mother, when it is suddenly seized with great dyspnœa, the face becomes blue, the eyes turn up, the hands are clenched, and without a cry the child drops dead. This affection of the thymus may develop rapidly, or in other cases the progress may be slower, and permit of a diagnosis being made and surgical interference adopted. Lange[6] has described a case of sudden death in an infant three and a half months old, from this cause. The trachea, one inch above the bifurcation, was contracted, so that the lumen, although not absolutely effaced, was like the hollow in the sheath of a sword. The thymus gland weighed over three-quarters of an ounce, and closely embraced and compressed the trachea.

**The Paralyses of Whooping Cough.**—This subject has been investigated by Dr. Charles Leroux,[7] who has collected 38 cases. The paralytic attack occurred most frequently during the acute stage of the disease, but sometimes during the declining stage. The various forms of paralysis were: (1) cerebral, commonly hemiplegia; (2) spinal; (3) peripheral, with involvement of different groups of muscles; (4) disseminated sclerosis, possibly antecedent to the whooping cough, but much aggravated by its development. Six out of the 38 patients died. The paralysis may be permanent. The morbid changes as revealed at several necropsies were congestions, hæmorrhages—meningeal, sub-meningeal, and cortical—and patches of inflammatory softening. He does not regard the violence of the cough as a sufficient explanation of these lesions, but believes that the poison of pertussis exercises a damaging influence on the coats of blood vessels in the nervous tissues, as happens in the case of other infectious diseases.

In the treatment of whooping cough Marfan[8] has found most benefit from the use of antipyrin, belladonna, and bromoform. In the case of belladonna it not infrequently happens that the physiological effects of the drug manifest themselves before the paroxysms of coughing show any diminution. Antipyrin is contraindicated if broncho-pneumonia is present owing to its depressing influence. Bromoform is not dangerous when used with ordinary precautions, and has no injurious effect on broncho-pneumonic patients. As to the general management, he holds that cases of whooping cough should not be allowed out of doors, but occupy two rooms, one by day and the other by night. If broncho-pneumonia develops the patient should be isolated from other cases of whooping cough, as a different infection has occurred.

[1] Birm. Med. Rev., May, 1893. [2] Berl. Klin. Wochensch, May 23, 1898; and Epit. Brit. Med. Jour., June 11, 1893. [3] Med. Rec., April 8, 1898. [4] Ibid., June 11, 1898. [5] Arch. f. Kinderheilk., B. 24, Heft. 2 and 3, S. 186. [6] Centralb. f. Gynecol., No. 20, 1898; and Epit. Brit. Med. Jour., July 16, 1898. [7] Jour. de Clin. et de Therap. Inf., Mar. and April, 1898; and the Lancet, May 14, 1898. [8] Jour. de Med., Mar. 10, 1898; and Epit. Brit. Med. Jour.; April 9, 1898.

